# Physical and Digital Infrastructure Readiness Index for Connected and Automated Vehicles

**DOI:** 10.3390/s22197315

**Published:** 2022-09-27

**Authors:** Boris Cucor, Tibor Petrov, Patrik Kamencay, Ghadir Pourhashem, Milan Dado

**Affiliations:** 1 Faculty of Electrical Engineering and Information Technology, University of Zilina, 010 26 Zilina, Slovakia; 2 Department of International Research Projects—ERAdiate+, University of Zilina, 010 26 Zilina, Slovakia

**Keywords:** cooperative, connected and automated mobility, infrastructure readiness assessment, connectivity data, positioning data, convolutional neural network

## Abstract

In this paper, we present an assessment framework that can be used to score segments of physical and digital infrastructure based on their features and readiness to expedite the deployment of Connected and Automated Vehicles (CAVs). We discuss the equipment and methodology applied for the collection and analysis of required data to score the infrastructure segments in an automated way. Moreover, we demonstrate how the proposed framework can be applied using data collected on a public transport route in the city of Zilina, Slovakia. We use two types of data to demonstrate the methodology of the assessment-connectivity and positioning data to assess the connectivity and localization performance provided by the infrastructure and image data for road signage detection using a Convolutional Neural Network (CNN). The core of the research is a dataset that can be used for further research work. We collected and analyzed data in two settings—an urban and suburban area. Despite the fact that the connectivity and positioning data were collected in different days and times, we found highly underserved areas along the investigated route. The main problem from the point of view of communication in the investigated area is the latency, which is an issue associated with infrastructure segments mainly located at intersections with heavy traffic or near various points of interest. The low accuracy of localization has been observed mainly in dense areas with large buildings and trees, which decrease the number of visible localization satellites. To address the problem of automated assessment of the traffic sign recognition precision, we proposed a CNN that achieved 99.7% precision.

## 1. Introduction

Connected and Automated Vehicles (CAVs) are expected to bring tremendous social, economic and environmental benefits, including increased road safety, addressing of road congestion and decreased environmental impact due to less wasted fuel thanks to improved vehicle management [1,2]. However, even the strongest supporters of the idea that vehicles should be as independent of the infrastructure as possible already accept the fact that automated driving can safely work only in specific Operational Design Domains (ODDs) [3]. Therefore, the physical and digital infrastructure already plays a role in the design and functioning of CAVs.

The preparation of infrastructure for automated driving is a multifaceted challenge, including components such as connectivity, provision of localization and mapping services, machine-readable road signage, and CAV-friendly road geometry and is a time-consuming, costly task requiring thorough planning. As highlighted in reference [4], automated driving functions relying on the infrastructure are often regarded from the perspective of a chicken-and-egg situation—infrastructure investments are postponed in an expectation that vehicle manufacturers take the lead and implement the related applications and vice versa. While Automated Driving Systems (ADS) are still under development, some of their basic requirements on the physical and digital infrastructure are already clear. Therefore, a complex assessment framework to help infrastructure providers evaluate the state of the infrastructure in terms of its readiness to support automated driving can be beneficial to help identify the essential areas of intervention and plan the investments and the timeline of infrastructure upgrades.

Artificial Intelligence (AI) is one of the key enabling technologies for the deployment of CAVs. Being an essential component responsible for a CAVs perception of the environment and decision making, application of AI in the context of CAVs has been a thoroughly investigated topic by both academia and industry. Interested readers are referred to the works of Ma et al. [5] and Li et al. [6] for thorough surveys on the state-of-the-art of implementing AI in CAVs.

Cybersecurity is another crucial aspect of CAVs operation that needs to be carefully considered. Ge et al. [7] proposed an algorithm to address the problem of resilient and safe platooning control of CAVs that are under denial-of-service attacks disrupting the V2V communication. Khan et al. [8] developed a conceptual system dynamics model to analyze the cybersecurity of CAVs. The model integrates six critical areas and their corresponding parameters that either enable or mitigate attacks on CAVs operation.

Several studies have already explored the key requirements on infrastructure for automated driving and assessed their impact on the performance of CAV operation. Carreras et al. [9] proposed a classification scheme to classify and harmonize the capabilities of a road infrastructure to support CAVs. Similarly to SAE levels of Driving Automation [10], the authors propose five levels of infrastructure support ranging from no support up to support sufficient to facilitate cooperative driving.

Mackenzie et al. [11] performed an assessment of line markings at multiple sites along Australia’s Great Southern Highway using two vehicles equipped with a lane departure warning system and two cameras. The authors conclude that the failure to accurately detect line marking crossing events can be most often attributed to the absence of a marked line, vehicle travel speed being lower than the speed recommended for system operation by the manufacturer, bad condition of lane markings and the low line marking retro-reflectivity and/or daylight brightness.

Magyari et al. [12] conducted a study on sight distances at unsignalized intersections, comparing the minimum required sight distances between automated and human-driven vehicles. The authors demonstrated that automated vehicles require 10–40 m shorter sight distances than conventional vehicles.

Liu et al. [13] identified infrastructure aspects that should be considered to be upgraded based on the gap between their current state and future requirements of CAVs. The authors concluded that the main infrastructure intervention areas that currently require attention should be traffic signs and road markings, communications, pavement structure and road surface, parking lots, service stations, safe harbor areas, roundabouts, bridges, drainage and geotechnical aspects.

Nitsche et al. [14] conducted a study about the requirements on road transport infrastructure for highly automated vehicles focusing on automation Levels 2–4. The methods used in the study consist of a literature review and an online survey with 54 multidisciplinary experts. The study identified the factors with the largest impact on the performance of ADS in three categories: lane assistance systems, collision avoidance systems and speed control systems. The authors also argued that the complex urban environments, temporary work zones and poor visibility due to bad weather conditions are the major infrastructure challenges for automated driving systems.

Madadi et al. [15] carried out an assessment of road network readiness based on a workshop including experts who judged images of specific infrastructure segments. Each of the two rounds of judgements was followed by a group discussion and a summary. The authors conclude that the experts identified many similar issues for different instances of roads and intersections.

Although there are numerous approaches already presented in the literature that aim at assessing the readiness of infrastructure for automated driving, those approaches are either limited to the assessment of a single performance indicator, do not provide measurable performance indicators at all, or rely on a per-segment evaluation performed by experts. Evaluation by a group of experts is both time-consuming and expensive for evaluating larger infrastructure networks. Moreover, it also brings the challenge of personal perception and subjective evaluation of readiness, as can be seen from often contradictory human assessments of the same infrastructure segment.

To allow large-scale, automated assessment of the physical and digital infrastructure readiness for automated driving, key aspects of the infrastructure contributing to the safe and reliable operation of CAVs and their minimum requirements in terms of measurable performance indicators have to be identified. Furthermore, a common methodology for the collection of required data, their processing and evaluation is essential to be developed, which, to the best of our knowledge, has not been presented in the available literature yet.

It is noteworthy to mention that the proposed assessment framework does not cover all the requirements of automated driving, as many of them are still under development. However, the framework provides a tool to infrastructure providers that can quickly and cheaply assess the extent to which the already known fundamental requirements are met and support them with identifying segments and specific interventions needed for increasing their readiness for automated mobility. The framework presented herein aspires to contribute toward solving the above-mentioned chicken-and-egg loop by providing a simple yet robust basis to identify the areas where infrastructure investments are necessary, regardless of the further developments in automated driving functions down the line.

The scientific and practical contribution of this paper can be summarized as follows:

- We propose a novel assessment framework to help infrastructure operators evaluate the readiness of physical and digital infrastructure for automated driving based on a set of indicators derived from the literature review on infrastructure requirements for CAV;
- We propose a data-collection setup and data processing methodology for collecting and evaluating data on the infrastructure necessary for the application of the assessment framework;
- We demonstrate the data collection and processing approach as well as the experimental results on a part of infrastructure in the city of Žilina, Slovakia.

The rest of the paper is organized as follows. The framework for assessment of physical and digital infrastructure for CAVs is described in Section 2. Section 3 contains a description of the data collection for connectivity, positioning and image data. The applied methods and methodology are described in Section 4. The achieved experimental results are presented in Section 5. Finally, Section 6 concludes by summarizing the results of this study, its contribution and further suggestions for future research.

## 2. Framework for Assessment of Physical and Digital Infrastructure Readiness for CAVs

In this section, we present the framework that we developed to assess the infrastructure readiness for Cooperative, Connected and Automated Mobility (CCAM). The framework is based on an extensive literature review of the requirements of CCAM, as well as on current industry best practices, anticipating the future requirements of various components of automated driving.

The framework aims to assess the infrastructure in five key areas crucial for CCAM implementation—connectivity, localization, machine-readable signage, and maps and object detection. For each area, a set of indicators is presented. A scoring grid mapping a score to an indicator value is assigned to each indicator. It is worth noting that the number of indicators selected for assessment of each area, as well as their corresponding assessment grids, may be subject to change and will be continually revised as new requirements for CCAM emerge or the currently identified ones are further clarified and quantified. The indicators for the current framework version has been selected in regard to the data that are either available now, or can be collected with a currently available technology. It should be noted that currently, it is not practical to provide an overarching index for each of the assessment areas since the level of impact of individual indicators on the performance of CAVs is not entirely clear yet. Therefore, the infrastructure assessment should be performed on a per-indicator basis.

The result of an assessment of an infrastructure segment is a numerical index, which represents the readiness of the evaluated road segment for CAVs deployment in the context of the corresponding indicator. This index can be useful for objective evaluation of the current status of infrastructure readiness and for planning and prioritizing future infrastructure upgrades, expansion and investments to increase its readiness to CAVs. The framework is presented in Table 1, Table 2, Table 3, Table 4 and Table 5.

### 2.1. Connectivity of Vehicles

It is already a well-accepted fact that connectivity and V2X communication will play a crucial role in the automation of road transport and addressing its safety challenges. A good example is a work by Zadobrischi et al. [16], who developed a system for analysis and management of dangerous situations that can detect a wide range of potentially hazardous conditions, including driver’s psychosomatic conditions, as well as attributes of nearby vehicles and pedestrians. The system communicates with various traffic safety elements through V2X radio frequency (RF) or Visible Light Communication (VLC). In order to share the detected hazards beyond the nearby connected vehicles in direct RF or VLC reach, a connectivity infrastructure based on either Dedicated Short-Range Communications (DSRC) Roadside Units (RSUs), or cellular networks has to be in place.

We would like to point out that the assessment framework for the connectivity area is technology agnostic, i.e., communication infrastructure based on any current (e.g., DSRC, 4G- or 5G-based Cellular-V2X, VLC), or future communication technology can be considered for V2X communication as long as it meets the corresponding performance indicators.

The values of indicators used to evaluate the connectivity area are based on the already identified requirements for V2X communications as well as on projected bandwidth needs for AVs. While the amount of data collected by SAE Level 5 AV’s sensors is expected to be huge [17], it is important to note that the majority of these raw measurements will be processed and utilized locally.

The lowest possible boundary of the bit rate has been set to 300 kbit/s. This bit rate corresponds to a connected vehicle broadcasting Cooperative Awareness Messages (CAM) with a maximum length of 1500 bytes while receiving CAMs from one neighboring vehicle at the same time with a message generation frequency of 10 Hz. If the infrastructure is not able to support this level of service, then no deployment of Cooperative ITS is possible, and therefore, the score of such an infrastructure segment would be equal to zero. On the contrary, if the 300 kbit/s bit rate is available for each vehicle at the given road segment, a score of 0.25 is awarded, indicating that at least a basic CAM service is available.

To achieve a score of 0.5, the infrastructure segment has to allow at least bi-directional sharing of sensory information on top of the basic CAM service. A sharing of footage from one automotive-grade camera with a resolution of 1280 × 1080 capturing a fairly complex scene, including buildings and vegetation, at 30 frames per second encoded by H.265 codec, including one 128 kbit/s audio track has been assumed. The resulting bit rate necessary to facilitate such traffic is 8.5 Mbit/s.

An infrastructure segment with a score of 0.75 is able to facilitate at least a bi-directional sensor sharing of one camera and a LIDAR sensor as well as a basic CAM service. From collected data, we empirically estimated the average bit rate of a 16-ray automotive LIDAR sensor to 7.66 Mbit/s. Therefore, the resulting minimum bit rate per vehicle necessary to achieve a score of 0.75 is 24 Mbit/s.

It is widely accepted that highly automated vehicles will utilize communication links with bit rates beyond 1 Gbit/s for extensive sensor sharing and operational data exchange. Therefore an infrastructure segment providing this level of service is awarded a score of 1.

To achieve the highest score in the message loss indicator, the communication infrastructure has to demonstrate at least 99.99% availability of service. On the contrary, if the average packet loss is above 10%, it might mean a steady information loss from more than one communicating vehicle, which, depending on the specific message content, might be unacceptable. Therefore, such a segment receives a score of zero.

The last evaluated indicator within the connectivity area is communication latency. The indicator values corresponding to the scores were derived from the networking and connectivity requirements of V2X communication services presented in [18].

### 2.2. Localization of Vehicles

Precise localization is a fundamental element of automated driving. Global Navigation Satellite Systems (GNSS) have become common tools to determine the precise location of vehicles and other road participants. Within the localization area of the framework, we evaluate four indicators (see Table 2) that provide insight into the availability and precision of the localization achievable by the GNSS at the given infrastructure segment.

Most GNSS techniques work with as few as five satellites. However, the redundancy is important for a number of reasons. First, the large number of satellites increases GNSS availability by providing service even if local obstructions block a significant part of the sky—a situation very common, especially in urban environments. Second, as demonstrated in [19], the performance of a three-constellation system, which sees only satellites more than 32 degrees above the horizon is equivalent to a single-constellation system in an open-sky scenario. Third, GNSS systems are developed independently, which allows performing cross-checking between constellations, enabling integrity guarantees [20]. Therefore, the average number of satellites as well as the number of available constellations a GNSS receiver can see when driving along the evaluated road segment are important indicators impacting the availability and performance of the localization service.

The values of the GNSS lateral localization error indicator corresponding to individual scores were derived using a methodology described in [21]. The lateral localization error below 0.1 m means that the vehicle is capable of determining its lane on a local road reliably (assuming a lane width of 3 m and a curvature of 20 m). If the lateral localization error is below 0.2 m, the vehicle is capable of determining its lane reliably on a highway (assuming a lane width of 3.6 m and speed of up to 137 km/h). If the lateral localization error is above 0.2 m, the vehicle might not be capable of determining its lane reliably.

It is worth noting here that the precision of the map also has to be factored in when evaluating the lateral localization precision. We assumed an equal error budget for the GNSS and the map.

### 2.3. Object Detection

The distance and reliability of object detection play a crucial role in automated driving as it is one of the key inputs into the CAV’s decision-making. Currently deployed CAVs use a multitude of sensors for object detection and advanced algorithms to classify those objects and assign them meaning. Most commonly used technologies use Light Detection and Ranging (LIDAR), Radio Detection and Ranging (RADAR) and camera sensors.

These sensors are usually embedded in the vehicle, but the infrastructure too can support the object detection either by transmitting information from sensors deployed along the road to the CAVs, hence extending their sensing capability beyond the onboard sensors’ line-of-sight, or by its geometry that accounts for the limitations of CAV sensing technologies.

An important attribute of the road infrastructure affecting the ability of CAV to detect other vehicles in time and prevent potentially dangerous situations, especially in an urban setting, is Intersection Sight Distance (SD). According to the methodology presented in the study [12], the required intersection sight distance along a major road SD for an automated combination truck can be computed as:

(1) $$\mathrm{SD}=0.278\;V_{d}t_{c}-\frac{V_{d}\left(t_{R1}-t_{R2}\right)}{3.6},$$

where $t_{c}$ is an acceptable time gap to enter the major road, $V_{d}$ is the design speed of the major road in km/h, $t_{R1}$ is the reaction time of a conventional vehicle in seconds, $t_{R2}$ is the reaction time of automated vehicle in seconds.

The critical gaps for various vehicle types (passenger car, single-unit truck, combination truck) and maneuver types (left turn, right turn, crossing) are provided in the American Association of State Highway and Transportation Officials Green Book [22]. From the road design point of view, we consider the worst-case scenario of a combination truck trying to perform a left turn at a STOP-controlled intersection. In such a scenario, the value of $t_{c}$ is 11.5 s.

Dixit [23] estimated the value of an automated vehicle’s reaction time to 0.8 s, while Guzek [24] provided the human driver’s reaction time on the brake pedal in the range between 1.2 and 2.2 s. We assume the difference in human-driven and automated vehicle’s reaction times of 1 s, a reasonable assumption commonly used in many studies, e.g., Schoettle [25]. However, it is worth noting that some studies, e.g., Rossi [26], point out that in the case when a driver has to take over the driving tasks from a Level 4 automated vehicle, the reaction time of the driver is, in fact, much larger than in the case of a manually driven vehicle. We do not consider this phenomenon for sight distance calculations.

In Table 3, we present the values of SD calculated for a passenger car, single-unit truck and combined truck for speeds of 50 and 90 km/h, which are the usual speed limits for urban roads and rural roads in Europe, respectively. It is worth noting here that the speed limit is usually set in the range of 80–90% of the road’s design speed. Therefore, *V*_d_ of 62.5 and 112.5 km/h was considered for computing the indicator values for urban and rural roads, respectively. A highway scenario has not been considered as the maneuvers performed on the highway are different from the ones performed on urban and rural roads, i.e., no left turns or crossings are allowed there.

The infrastructure segment that satisfies the SD criteria for the combination truck is assigned a score of 1 in the framework. An intersection that satisfies the criteria for a single-unit truck is assigned a score of 0.75. An intersection that satisfies the minimum SD criteria for a passenger car is assigned a score of only 0.25 since its ability to provide safe maneuvering space to any larger vehicle type than a passenger car might be severely limited.

The indicator “Infrastructure for remote sensor sharing available” refers to the ability of the infrastructure to sense the traffic situation at the assessed road segment and share its sensor data with the CAVs heading to that segment before they are able to detect the situation with their onboard sensors. An example might be a pedestrian crossing equipped with a radar sensor and Infrastructure-to-Vehicle communication capability to share information about the presence of pedestrians in an area where the CAV’s sensor detection range might be limited due to obstacles or road geometry.

### 2.4. Quality of Maps

Just as conventional vehicles, CAVs use outdoor structured roads whose basic attributes such as location and geometry are a priori known. These static road data can be pre-created and provided to the vehicle. In combination with GNSS, inertial navigation and odometry allow the vehicle to perform high-precision (centimeter level) positioning in real-time, reducing the complexity and cost of the CAV’s systems significantly [27]. Once the vehicle establishes its precise position on the road, it can use the a priori information from the map to make decisions about maneuvers and navigation, some of which would not be possible relying only on the sensor-based road model recognition methods [28]. Therefore, high-precision maps are considered one of the core enabling technologies for automated driving.

Obviously, the available maps come with different levels of precision and provide different richness of additional information about the infrastructure, ranging from the provision of basic static data on road geometry to highly dynamic high-definition maps updated in real-time and reflecting the current traffic situation.

Table 4 presents the indicator for the Quality of maps framework area with suggested map attributes and corresponding scores.

It is worth noting here that for the purpose of assessment, the score of a road segment without a fully updated map should correspond to the real state at the time of the data collection, i.e., if the traffic signs on the map are not up-to-date, the segment should be scored as there were no traffic signs on the map available at all.

### 2.5. Machine-Readable Signage

Road segments where either no high-definition map is available or where a mixed traffic of conventional and CAV traffic is expected, CAVs need to detect and recognize road signage using their own sensors. Numerous studies, e.g., [29,30], conclude that the features of road markings that are key for their recognition by human drivers, such as retroreflectivity and contrast, are also important in the case of marking detection by CAVs.

Table 5 presents the assessment framework and indicators proposed for the Machine-readable signage area of infrastructure assessment.

We consider two indicators within this assessment area—precision of horizontal signage detection and precision of vertical signage detection by an automated detection system. We detail the CNN used to evaluate the precision of vertical signage detection indicator from the collected sample data in Section 4.2. In this article, we will consider only vertical marking (vertical signs). Road traffic participants will also be included in the neural network training process.

Waykole et al., in [31], conducted an extensive literature review on lane detection and tracking algorithms for advanced driver assistance systems. The authors conclude that the lane detection and tracking efficiency rate under dry and light rain conditions is near 99% in most scenarios. Therefore, we adopt this value of precision for the infrastructure segment to be scored by a score of 1. To achieve a score of 0.5, a segment of infrastructure has to provide road markings clear enough to allow precision of detection in the range between 90% and 99%, which is equivalent to a precision of a lane detection and tracking system operating during the night at isolated highways. When the precision of horizontal signage detection is between 80% and 90%, the infrastructure markings only provide a performance equivalent to a vanishing point detection system operating on unstructured roads. Such an infrastructure segment is awarded a score of 0.25.

Due to the high variation in detection results in different testing environments, the evaluation of the precision of horizontal signage detection is a complex problem on its own, requiring an extensive definition of test scenarios, which is out of the scope of this paper. Therefore, we refer the interested reader to the relevant works summarized in [31] and relevant automotive standards such as [32] for further details on measurement methodology and test settings.

## 3. Data Collection and Processing

In this section, we interpret the technical parameters of used data systems and describe the parameters of positioning and the connectivity dataset. We also describe the system for the collection of image data.

### 3.1. Connectivity and Positioning Data

**Figure 1:**
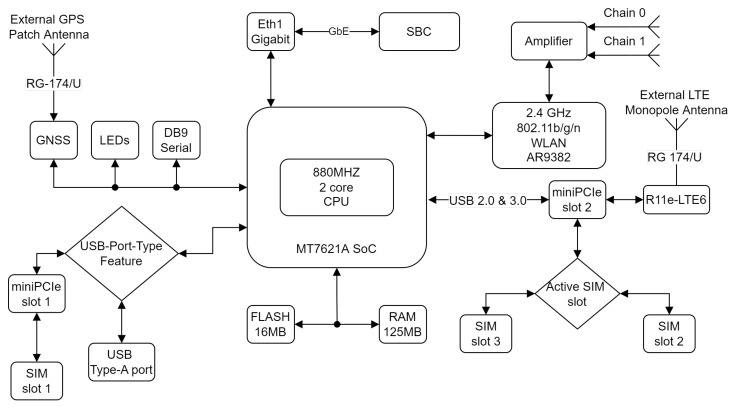
Collection system block diagram [33].

To collect the connectivity and positioning data, a Mikrotik LtAP LTE6 wireless access point and a Single-Board Computer (SBC) were used. LtAP LTE6 is a compact wireless access point with built-in GPS. LTE connectivity was enabled by the Mikrotik R11e-LTE6 LTE modem connected to miniPCIE slot integrated in the Mikrotik LtAP LTE6 access point, as shown in Figure 1. The used LTE modem belongs to the LTE CAT6 category and provides a maximum download speed of 300 Mb/s and an upload speed of 50 Mb/s. The collection system block diagram is shown in Figure 1.

**Figure 2:**
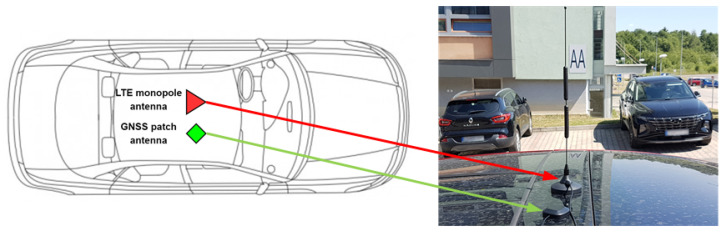
Antenna placement view.

For LTE transmission, an external 3dB wideband monopole LTE antenna with a resonant frequency from 698–2690 MHz and an input impedance of 50 Ω was used. For GPS reception, a Mikrotik ACGPSA external 26 dB, 50 Ω antenna with a resonant frequency of 1575.4 MHz was used. To ensure effective communication, we installed both antennas on the top of the testing vehicle’s roof, as illustrated in Figure 2.

#### 3.1.1. Positioning Data

**Figure 3:**
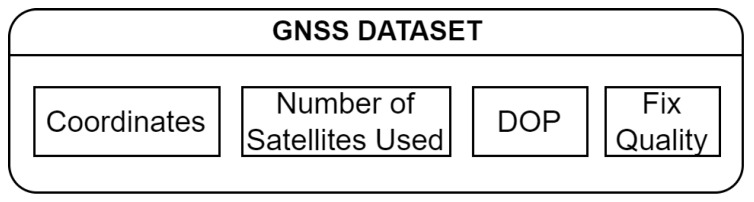
Block scheme of the position dataset.

The used device supports GPS, GLONASS, BeiDou and Galileo GNSS standards. The following telemetry of GNSS was collected as is shown in Figure 3—GPS coordinates, number of satellites used, Dilution of Precision (DOP) and fix quality.

The Dilution of Precision (DOP) is an important factor in determining positional errors in a GPS system. It is the collection of satellites’ geometry constellation from which signals are actually received. Basically, four satellites are the minimum required value to determine a complete positional fix in three dimensions. DOP is calculated using geometrical correlations between the position of the GPS receiver and the positions of the GPS satellites. The exact locations of these satellites relative to the receiver have an effect on the positional error. If the GPS receiver communicates with satellites spread throughout the sky, the calculated position will be more accurate, and the DOP value will be low. However, when satellites are close to each other, the calculated position will be less accurate, and the DOP value will be high [34,35]. In the following table, the DOP value rating is shown (Table 6).

The following metrics are used to describe DOP. Position Dilution of Precision (PDOP), Horizontal Dilution of Precision (HDOP), Vertical Dilution of Precision (VDOP) and Time Dilution of Precision (TDOP).

PDOP describes the number of satellites used that are spread in the sky. The more satellites directly above GPS receiver are used, the lower the PDOP value is. The effect of DOP on the horizontal position is described by HDOP. The HDOP and horizontal position (latitude and longitude) are better when more GPS satellites are used. The effect of DOP on the vertical (altitude) position is referred to as VDOP. The time difference between the GPS satellites’ and the GPS receiver’s internal clocks are represented by TDOP. A low TDOP value represents more accurate time synchronization. Because DOP metrics are derived from convergence, they are not independent. For example, a high TDOP value represents worse clock synchronization, and it has an effect on positional error [34,36,37].

The type of signal or technique used by the GPS receiver to establish its location is represented by the GPS fix status telemetry. The number of GPS satellites and techniques used by the GPS receiver are used to determine the GPS fix type technique. In general, the fix quality rating is given by numbers ranging from one to five, as Table 7 shows. The fix quality number represents the type of GPS technique that was used to determine location. Each technique has a different accuracy. The used GPS technique can be GPSFix, Differential GPS (DGPS), Precise Positioning System (PPSFix), Fixed Real Time Kinematic (RTK Fixed) or Float Real Time Kinematic (RTK Float). The GPSFix describes a basic GPS technique or Standard Positioning Service (SPS). SPS is a standard service provided to any user worldwide, without qualification or restrictions. Based on US security interests, the accuracy of this service is determined by the US Department of Defense [34,37]. Unlike GPSFix, the DGPS technique utilizes a network of ground stations used to broadcast the divergence between indicated position by GPS satellites and the real known position. PPSFix stands for most precise localization technique provided by GPS. Only the Federal Government and military have access to this service, which is encrypted. The RTK Fixed technique is used to optimize position accuracy calculated by DGPS. The technique is based on carrier phase measurement of the GPS, GLONASS, Galileo. Thanks to high accuracy, this technique is used for geodetic measurement purposes. On the other hand, RTK Float is a similar technique as RTK Fixed but with low accuracy. The accuracy is decreased by skipping the phase initialization process, which increases the speed of position calculation [34,38,39].

#### 3.1.2. Connectivity Data

**Figure 4:**
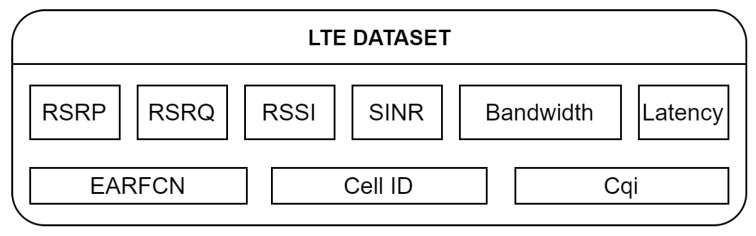
Block scheme of the connectivity dataset.

The following telemetry of LTE communication was collected, as is shown in Figure 4. Communication latency, bandwidth, Signal Interference Noise Ratio (*SINR*), Received Signal Strength Indicator (*RSSI*), Reference Signal Received Quality (*RSRQ*), Reference Signal Received Power (*RSRP*), E-UTRA Absolute Radio Frequency Channel Number (*EARFCN*), Cell Identification (Cell ID), Channel Quality Indicator (*CQI*) and Rank Indicator (Ri).

**Figure 5:**
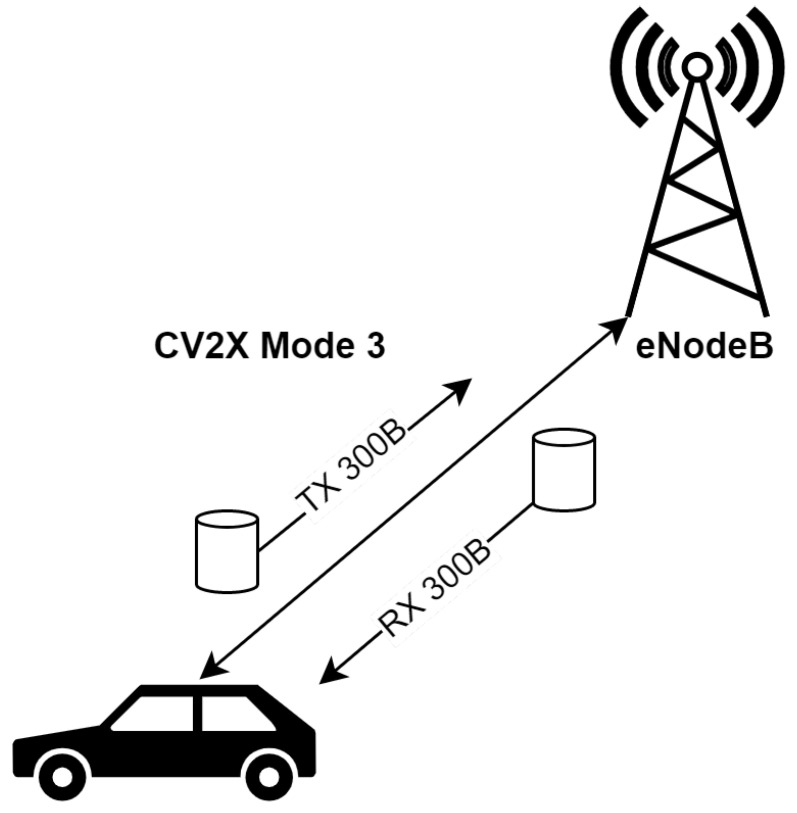
Emulated CV2X Mode 3 communication scheme.

Our priority was to emulate the Cellular-V2X (C-V2X), as there is currently no DSRC- and VLC-enabled communication infrastructure available along the investigated route. We set up SBC to send communication packets periodically to a virtual server. We chose a packet length of 300 bytes and period of 100 ms since these values are commonly used to represent a transmission of CAM [40]. The virtual server re-sent the packet back to SBC, as is shown in Figure 5.

Each sent and received packet was marked with a time stamp by Network Time Protocol. The two-way latency communication (Δt) was calculated depending on packet transmit time $\left(t_{tx}\right)$ and packet received time $\left(t_{rx}\right)$, as shown in equation:

(2) $$\Delta t\left[ms\right]=t_{rx}-t_{tx}\;.$$

Signal Interference Noise Ratio (*SINR*) represents the signal quality based on the strength of the wanted signal compared to the unwanted interference and noise. The *SINR* is a metric used in cellular networks to determine if a particular frequency resource is acceptable for maintaining a communication link. The network employs *SINR* to track radio link and handover failures. In systems that employ multiple access technologies based on frequency division, the scheduler can take *SINR* into account while allocating frequency resources [41]. It is a signal quality metric that is established by the User Equipment (UE) manufacturer instead of the 3GPP specifications. The basic *SINR* mathematical expression is shown in Equation (3):

(3) $$SINR\left[dB\right]=\frac{S}{I+N}\;,$$

where *S* stands for the strength of the usable signals. *I* stands for interference power of signals or channel interference signals from other cells. *N* stands for background noise, which is proportional to measurement bandwidths and receiver noise coefficients. Table 8 shows the standard *SINR* values and signal quality category.

Higher *SINR* values can affect the spectral efficiency as it enables the receiver to decode a higher Modulation Coding Scheme (MCS). To provide the best possible User Experience, the network operator attempts to optimize *SINR* at all locations, either by transmitting at a greater power or by avoiding interference and noise [43].

*SINR* optimization can aid in achieving higher cell capacity by allowing higher QAM modulation, which results in greater peak data rates, fewer missed calls, and an overall better quality of user experience [44].

Received Signal Strength Indicator (*RSSI*) is an LTE metric that states how much overall wideband power measured in symbols have been received, including all interference and thermal noise. UE does not send *RSSI* values to eNodeB. It may be easily calculated using *RSRQ* and *RSRP*, which are instead reported by UE. The value is measured in dBm. *RSSI* is defined as [45]:

(4) RSSI[dBm]=S+I+N,

where *S*, *I* and *N* are the same parameters as in the *SINR* equation.

**Figure 6:**
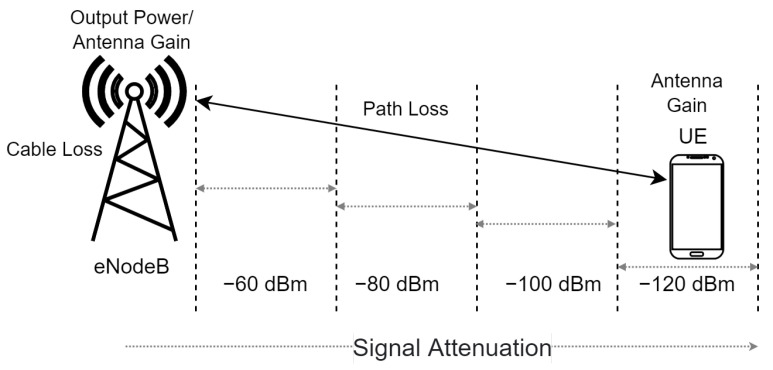
*RSRP* measurement.

Reference Signal Received Power (*RSRP*) and Refence Signal Received Quality (*RSRQ*) are two main key signal level and quality indicators for current LTE networks. When a UE goes from cell to cell in a cellular network and conducts handover, it performs a measurement of the reference signal strength and quality of serving and neighbor cells for successful execution of the handover process. In essence, it is the power of the received signal from eNodeB by UE [42]. Based on *RSRP*, it is possible to compare the strengths of signals from individual cells in LTE networks. The measurement process is shown in Figure 6.

Figure 6 shows eNodeB and UE, which receive the reference signal from the eNodeB. The closer the UE is to the eNodeB location, the stronger the received signal. The reporting range of *RSRP* is defined from −140 to −44 dBm with 1 dB resolution [42]. Table 9 shows the standard *RSRP* values and signal quality category.

The *RSRP* calculation is shown in Equation (5), where *N* stands for Number of PRBs (Physical Resource Blocks) [42,47,48].

(5) RSRP[dBm]=RSSI−10∗log(12∗N).

*RSRQ* telemetry parameter is the proportion of *RSRP* to wideband power. *RSRQ* represents signal quality received by the UE. The signal, noise, and interference received by the UE also have an effect on the *RSRQ* [40,42]. The following equation [42] is used for *RSRQ* calculation, where *N* stands for Number of Physical Resource Blocks (PRBs).

(6) RSRQ[dB]=N∗RSRP/RSSI,

The reporting range of *RSRQ* is defined from −3 to −20 dB. Table 10 shows the standard *RSRQ* values and signal quality category.

Instead of reporting raw carrier frequencies in MHz, LTE base stations use the ETSI E-UTRA Absolute Radio Frequency Channel Number (EARFCN) industry standard to report channel numbers. In LTE technology, EARFCN determines the carrier frequency in the uplink and downlink, the range of which is from 0 to 65,535. The equations below express the relationship between EARFCN and its uplink/downlink carrier frequency [49].

(7) $$F_{downlink}=F_{\mathrm{DL-low}}+0.1\left(N_{DL}-N_{\mathrm{offs-DL}}\right),$$

(8) $$F_{uplink}=F_{\mathrm{UL-low}}+0.1\left(N_{UL}-N_{\mathrm{offs-UL}}\right),$$

where *N*_DL_ stands for downlink EARFCN, *N*_UL_ for uplink EARFCN, *N*_offs-UL_ offset used to calculate uplink EARFCN, *N*_offs-DL_ offset used to calculate downlink EARFCN. The values *F*_UL-low_, *F*_DL-low_, *N*_DL_, *N*_UL_, *N*_offs-DL_, *N*_offs-UL_ are given in [49] by ETSI.

**Figure 7:**
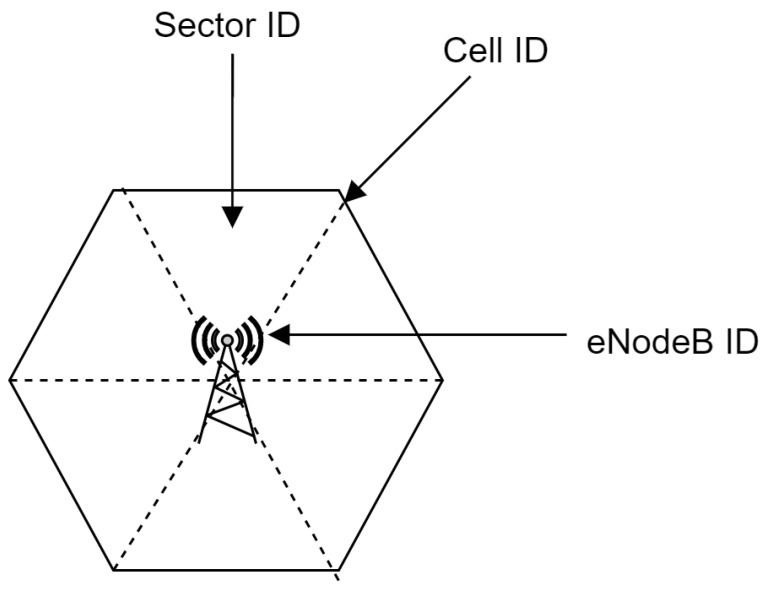
Description of the E-UTRAN identifiers.

For unique identification of LTE components, the identification numbers are used. As shown in Figure 7, we have three main key identifiers in the LTE cell. The E-UTRAN Cell Identifier (ECI) represents the identity of a cell within a Public Land Mobile Network Identifier (PLMN). ECI consists of 28 bits where the first 20 bits represent the eNodeB ID number and the last 8 bits are stated for cell ID. The sector ID identifies a particular antenna in cell sectors [50,51].

The code rate and modulation are defined by MCS in the LTE. MCS specifies the maximum number of usable bits that can be transferred per Resource Element (RE), and it is affected by radio channel quality. Table 11 shows the CQI-MCS mapping for LTE rel. 12 and beyond. The better channel quality is represented by a higher MCS, and the more useful data can be transmitted. In other words, MCS depends on error probability. In LTE, a Turbo encoder with a 1/3 coding rate is employed. The actual ratio of usable bits to total transmitted bits (useful bits + parity bits) is dependent on the quality of the radio link. The range of coding rates is 0.0762 to 0.9258.

Radio link quality is estimated based on the Channel Quality Indicator (*CQI*). The *CQI* parameter is reported by UE to the eNodeB. The *CQI* measurement is based on the Cell Reference Signal (CRS) [52]. Better radio condition is represented by higher *CQI* and the higher coding rate, as is shown in the table below. Bits per RE column should be multiplied by the number of data streams to obtain a final value in case of MIMO usage [52].

#### 3.1.3. Collection of Image Data

For image data collection, the OmniVision OV10640 camera system (OmniVision, Santa Clara, CA, USA) was used. This sensor uses a proprietary technology, which delivers an image with a very high dynamic range (HDR). Furthermore, the sensor is encapsulated in a compact package, which can be easily deployed for a wide range of automotive applications (see Table 12). A total of four cameras were used on the bus (one on the windshield recorded the area in front of the bus, one on the rear window recorded the area behind the bus, and one on each side recorded the area on the sides of the bus).

**Figure 8:**
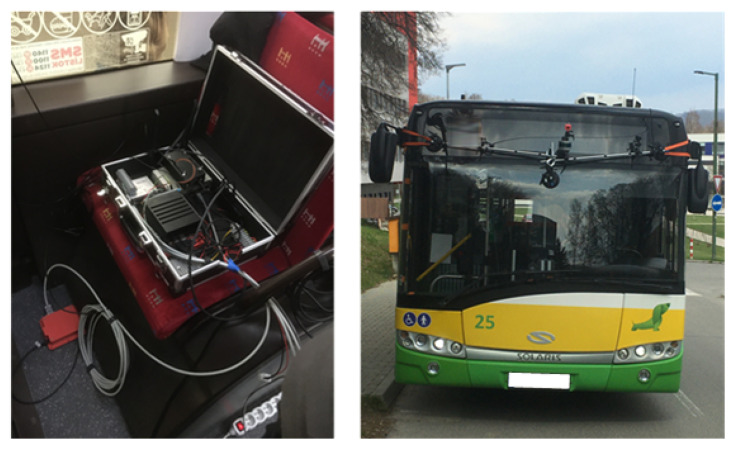
Image data collection.

Three cameras were used in the collection of image data. Two cameras were placed on the sides of the bus and one in the middle of the bus above the windshield (see Figure 8). The cameras located on the sides of the bus had a standard horizontal field of view (52 degrees). The middle camera capturing objects in front of the bus was a fisheye (horizontal field of view of 194 degrees).

Furthermore, the sensor is capable of sampling the recorded scene simultaneously instead of sequentially, which helps to minimize the distortion caused by motion.

**Figure 9:**
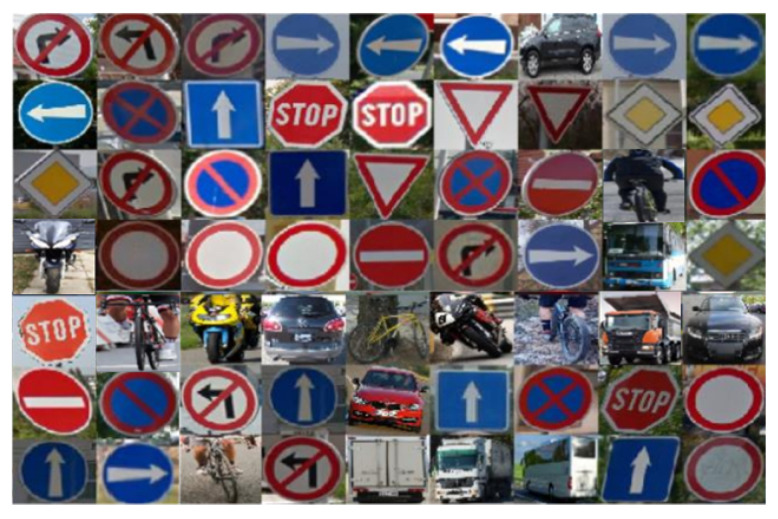
An example of an image dataset.

The image dataset contains classes representing traffic signs and also classes representing road users, as is shown in Figure 9. Table 13 shows all the classes that our image dataset contains. The first column contains the number of classes, and the second column shows the specification of the given class.

## 4. Methodology

In this section, we describe the processing of connectivity and positioning data and processing of image data using the proposed CNN.

### 4.1. Processing of Connectivity and Positioning Data

**Figure 10:**
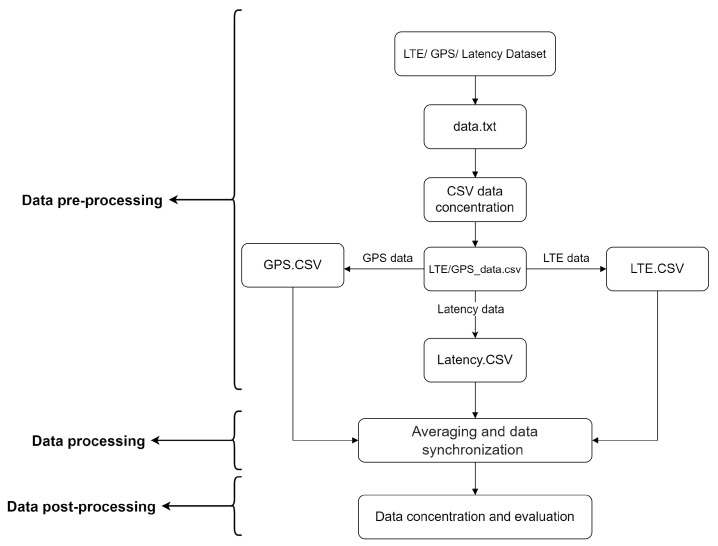
Data processing block diagram.

The data collection process was repeated four times on different days and at different time. Data processing was divided into three parts, as illustrated in Figure 10. In the first part, data pre-processing, the data were prepared for processing. Data were collected as text files (.txt), and it was necessary to convert them to Comma-Separated Values (CSV) and separate them. The conversion process and data separation was performed by a python script.

The pre-processed data served as an input to the processing stage. In this stage, the latency data were averaged because latency was measured every 100 ms, and other data were collected every 1000 ms. After the averaging process, all data were synchronized on the basis of a time stamp that was obtained via Network Time Protocol (NTP) during the data collection. A weighting coefficient was assigned to examine parameters on the basis of which it is possible to represent the quality of the digital infrastructure parameters. In the data post-processing stage, data were concentrated, evaluated, and visualized.

### 4.2. Processing of Image Data Using CNN

**Figure 11:**
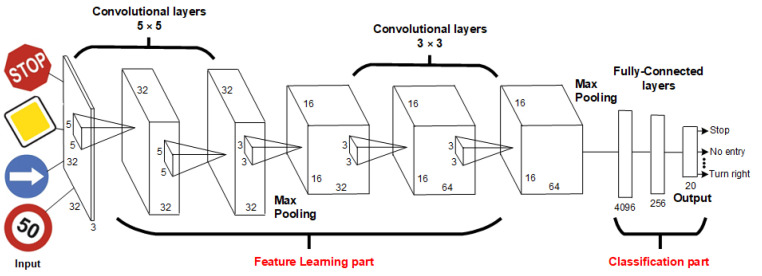
Proposed architecture of CNN.

For traffic sign recognition, we proposed CNN, which is detailed in Figure 11 and Table 14. We selected CNN since, depending on the used hardware, it has a potential to process data in real-time. Hence, it can serve as a basis for the future development of an automated infrastructure readiness assessment system operating in real-time.

The CNN is divided into two parts, the feature learning part (convolutional part) and the classification part. The convolution part is used for data processing. The classification part serves to transform the format of the processed data and to classify the output. Figure 11 shows the block diagram of the proposed CNN. This proposed CNN consists of 12 layers (four 2-D convolutional layers, two MaxPooling layers, three layers for turning off neurons (Dropout) and 3 fully connected layers.

As discussed in [53], the image data that are corrupted by various noises impact the resulting performance of the proposed neural network. For this reason, the noisy image data are recovered with the pre-processing step (using various filters). This step improves the overall performance of the proposed neural network.

The first and input layer is the convolution layer. The input is represented by images with dimensions of 32 × 32 pixels. At the input of the layer, we will therefore have 32 × 32 neurons (1024 arranged in a square matrix). Each pixel in the image is represented by an 8-bit number, ranging from 0–255 for each color. Sometimes it also uses a black and white image, which is represented by one channel in the same range, where 0 represents white and 255 black. In our case, each pixel is represented by three values from the RGB palette. Together, these values form three two-dimensional matrices, which together form the image volume. In this layer, we use 32 filters with dimensions of 5 × 5. We also use the padding parameter set to “Same”, which will cause the output feature maps to be the same size as the input image. The output from this layer will be 32 (32 × 32 feature maps for each input image).

The second layer is the convolutional layer, which includes 32 feature maps with dimensions of 32 × 32. It contains 32 filters with a window size of 5 × 5. It also contains a parameter that maintains the same dimensions of the output as the input. The output of the layer will be 32 × 32 × 32 feature maps for each image. The third layer is a merging layer (MaxPooling layer) with a filter size of 2 × 2 and a maximum value criterion. This causes the dimensions of the 32 × 32 × 32 input features to be halved in the output. The number of feature maps remains the same. In the end, we obtain 32 feature maps with dimensions 16 × 16. The fourth layer is dropout with a parameter of 0.5, which means that 50% of the random neurons at the input will be turned off. This layer preserves the previous dimensions of the output. The fifth and sixth layers are convolutional, which includes 32 feature maps with dimensions 16 × 16 and 64 filters with dimensions 3 × 3, respectively. The seventh layer is a merging layer with a filter size of 2 × 2 and a maximum value criterion. The dimensions of the output will be twice as small as the input, i.e., 64 × 8 × 8 with 64 feature maps. The eighth layer is a dropout with a parameter value of 0.5, which means that 50% of the random neurons on the current layer will be turned off. The ninth layer is flattened, which transforms feature maps into fully connected layers. This layer will contain 8 × 8 × 64 neurons, which is a total of 4096 neurons and, therefore, also 4096 outputs. The tenth layer is Dense, which represents the classic fully connected layer. It contains 256 neurons. The last layer is the Dense layer, which classifies the output from the network. It contains 20 neurons (20 classes). Each neuron represents a given class. In this layer, we use the sigmoid activation function, which classifies us with the probability that a given neuron is activated, thus determining to which class it belongs.

## 5. Experimental Results

In this section, the performance evaluation of the proposed method based on the created dataset is discussed.

### 5.1. Results for Connectivity and Positioning Data

**Figure 12:**
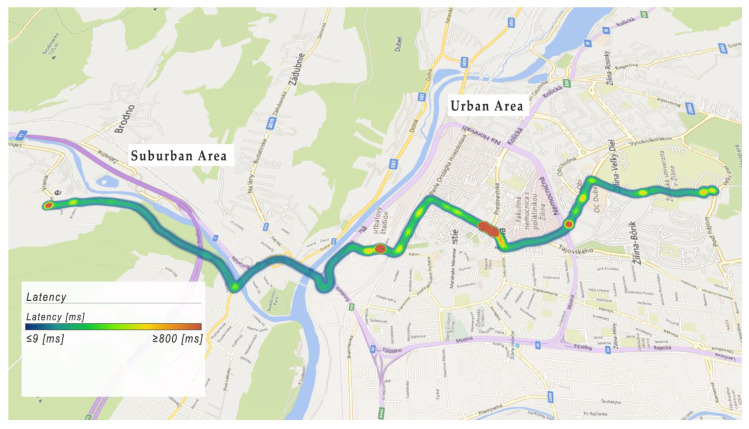
Example of latency data collection.

Analyzed connectivity data are interpreted on the maps with the heat map route, which shows the analyzed route in the city. Figure 12 shows the communication latency on the analyzed route. The blue color represents the latency values less than or equal to 9 ms, and the red color represents values greater than or equal to 800 ms.

As we can see, the latency value is higher in urban areas than in suburban areas. The average latency value in urban areas was 83 ms, and in a suburban area, 42 ms. We found spots where the latency was higher than 800 ms in an urban area. These places are interpreted by red color, and they are located mainly at intersections with heavy traffic or near points of interest. The latency value in these places reached the value of 1500 ms. For the deployment of CCAM, it is necessary to support telecommunication infrastructure in these red areas. Please note that during the data collection campaign, no considerable traffic jams or road congestions occurred along the investigated route. In the case of extremely congested traffic, the communication performance is expected to drop even further.

**Figure 13:**
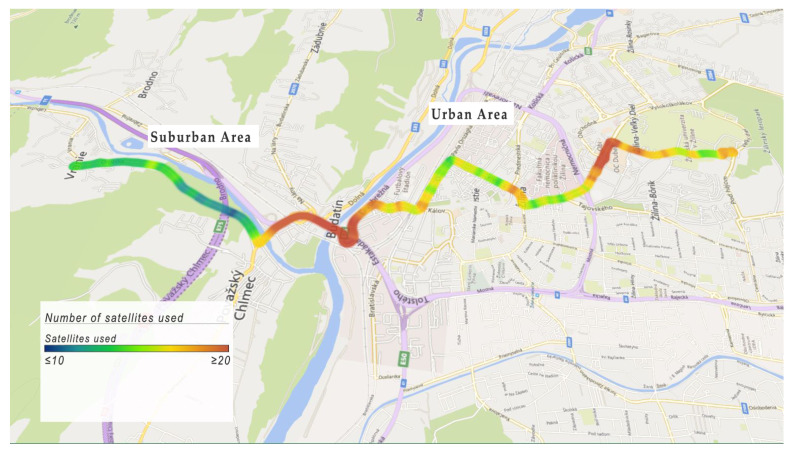
Example of satellite numbers used.

The number of GNSS satellites used, i.e., the number of visible satellites, ranged from 14 to 22. The maximum number of visible satellites was reached in sparsely-built areas with a clear vision of the sky. In Figure 13, the blue color represents the usage of less than or equal to 10 satellites. The red color represents the usage of more than or equal to 20 satellites. The number of visible satellites was lower in dense urban areas and in the suburban area too.

**Figure 14:**
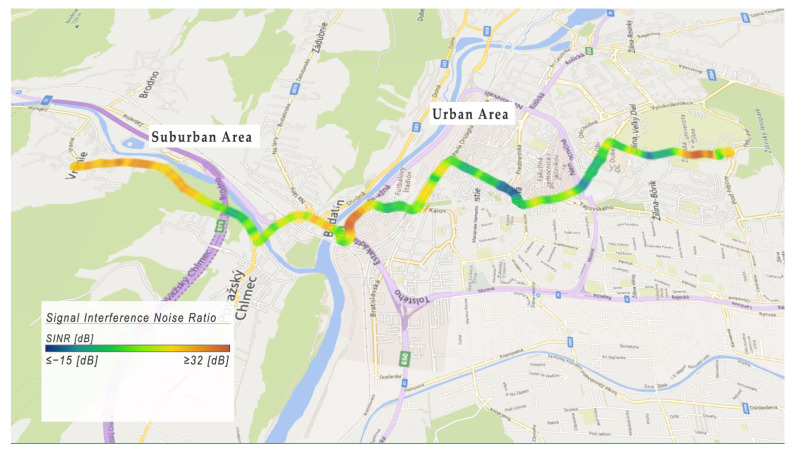
Example of Signal Interference Noise Ratio.

The results for the Signal Interference Noise Ratio are shown in Figure 14. This map interprets the coverage quality of the 4G telecommunication infrastructure. The blue color represents *SINR* values less than or equal to −15 dB, and the red color represents *SINR* values greater than or equal to 32 dB. As shown in Figure 14, the better coverage is in the suburban area compared to the urban area. In the urban area, there were spots in which the *SINR* value was −15 dB, which is also related to poor connectivity parameters.

**Figure 15:**
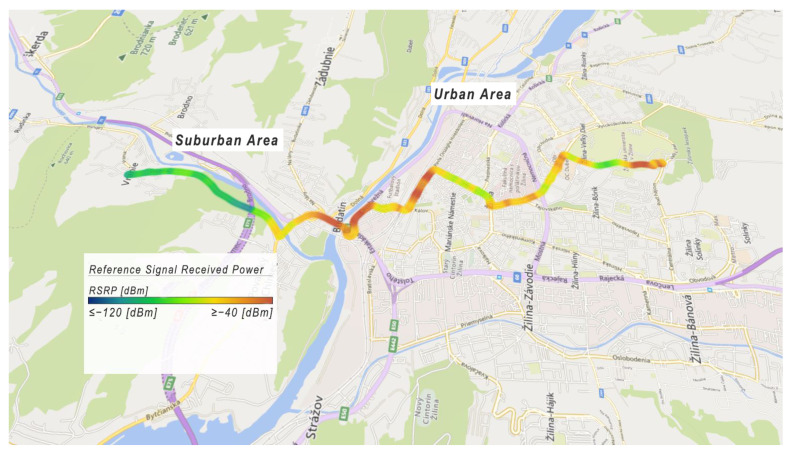
Example of Reference Signal Received Power.

As can be seen in Figure 15, *RSRP* values less than or equal to −120 dBm are represented by blue. *RSRP* values higher or equal to −40 dBm are represented by red. The received power of the reference signal is lower in the suburban area compared to the urban area. It is caused by the fact that there are many eNodeBs in the city, which ensure handover and thus provide higher power of the reference signal. On the other hand, in the suburban area, there is a low number of eNodeBs, which reduces the received power of the reference signal.

**Figure 16:**
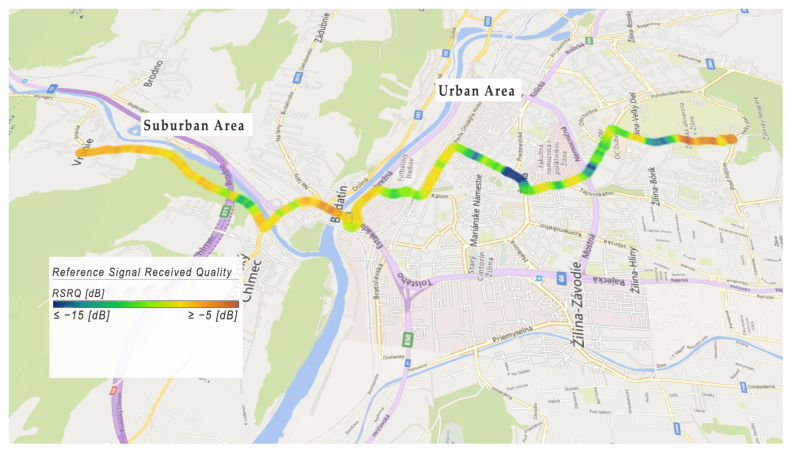
Example of Reference Signal Received Quality.

The received quality of the reference signal ranges from −15 to −5 (dB). As can be seen in Figure 16, in an urban area at an intersection with heavy traffic, the received quality of reference signal is lower than in other areas. The blue color represents an *RSRQ* value lower than or equal to −15 dB, and the red color represents an *RSRQ* value greater than or equal to −5 dB.

Fades in received reference signal power are caused by larger communication distance and resulting lower signal power from the individual cells in the 4G network, which is spread over the area. Near the eNodeBs, the quality of the received reference signal was −5 (dB), which represents a better condition of the connection.

### 5.2. Results for Image Data

The inputs to the proposed CNN were image data of size 32 × 32 × 3. The model was trained on 30 epochs. In the training process, it is important to find the point where the network gives us the best results. If we exceed this threshold, the network learns too much detail, which means that the success on the validation or test model decreases (overtraining of neural network). On the other hand, if we stop learning too early, the network will be untrained. For this reason, we use checkpoints and also dropout layers, which improve our model. In the figure below, can be seen how we split the data into train, validation, and testing. In our work, we use this proportion, but it can be changed as per the requirement. To build CNN for traffic sign classification, the Keras deep learning framework was used.

**Figure 17:**
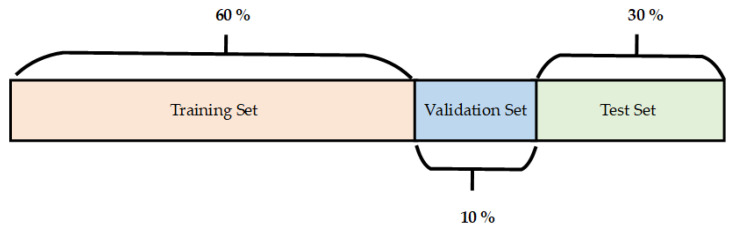
Division of the image dataset (training data, test data and validation data).

Each class contains 1670 images. The dataset was divided into training, testing and validation parts in the ratio 60:30:10, as is shown in Figure 17. This means that 60% of images were used for training data, 10% for validation data and 30% for test data. The size of each animal image was 32 × 32 pixels.

**Figure 18:**
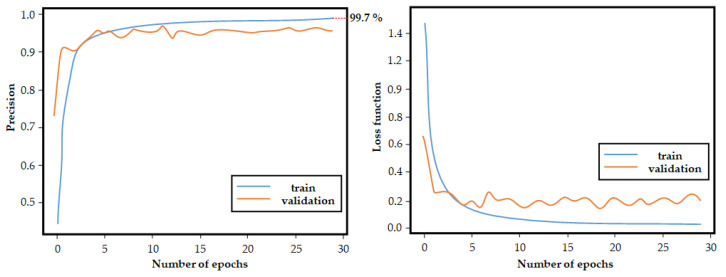
Training and validation precision.

The training set and the validation set were used, respectively, to train and optimize the model. The test set was used to check how the model performs on unseen data. As can be seen in Figure 18, precision of 99.7% on our training set (blue line) was obtained. Please note that this precision is very similar to the results presented by other works, such as [54,55].

**Figure 19:**
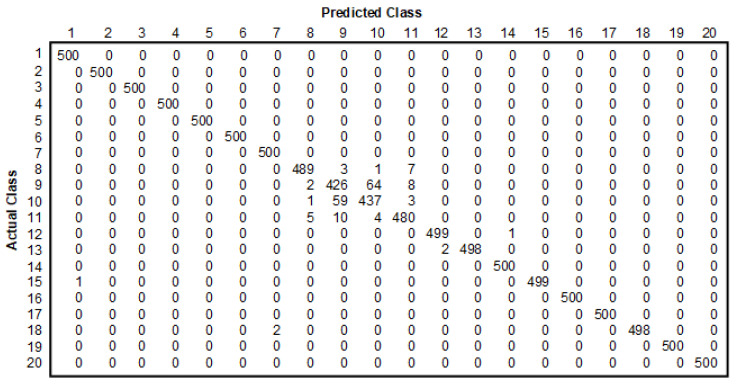
Confusion matrix.

Figure 19 demonstrates the confusion matrix. The rows of the confusion matrix represent the actual class, while the columns of the confusion matrix represent the predicted class. The values along the main diagonal represent images that correctly classified images to be the same class. The correctly classified images across all classes are used to define the classification accuracy. In other words, it is the ratio of the sum of the correctly labeled images to the total number of images in the test dataset. In the case of traffic sign classification, the precision was greater than 99%. On the other hand, in the case of the classification of traffic participants, the precision was 98.4% for pedestrians, 85.5% for cyclists, 86.4% for motorbikes and 96.4% for scooters (see Table 15).

## 6. Discussion and Conclusions

The result of our research is a framework that serves to assess the state of physical and digital infrastructure readiness for CCAM. The core of the research is a dataset that can be employed for further research on the topic. Our results can serve as a basis for more effective planning of infrastructure development from the point of view of readiness for CCAM. The main goal of the connectivity and positioning data metering is research on the performance of new generation networks and localization systems for CCAM readiness. As part of this research, we mapped and analyzed the urban and suburban areas. Despite the fact that the analysis and mapping were carried out in different time frames and days, we found underdimensioned areas on the investigated route. The main problem of data communication analysis is the latency. As we described in Section 5.1, we found the critical places in urban areas from the point of view of latency. These places are mainly located at intersections with heavy traffic or near points of interest. A possible solution to high latency is to add microcells to critical places. On the one hand, adding microcells can not only lower the latency, but it can also increase the performance of telecommunication networks and increase coverage. On the other hand, this solution comes with increased infrastructure costs. The best solution for accelerating the implementation of CCAM is the deployment of 5G networks. Currently, 5G networks are not widely deployed, and they are mainly located in metropolitan areas and capital cities in some designated locations. Analysis of the performance of the 5G network from the point of view of CCAM readiness will be our future work. The issue of localization is dense areas with large buildings and trees. It affects the number of visible satellites, which also has an impact on the accuracy of localization. A possible solution is to use 5G networks in conjunction with GNSS. This can improve the better localization accuracy in dense urban areas. Using GPS, digital maps and neural networks, the vehicle can recognize the direction of travel, speed, lane detection and traffic signs. By combining neural network, GPS data and digital maps, it is possible to create a reliable system that could reliably recognize traffic signs.

**Figure 20:**
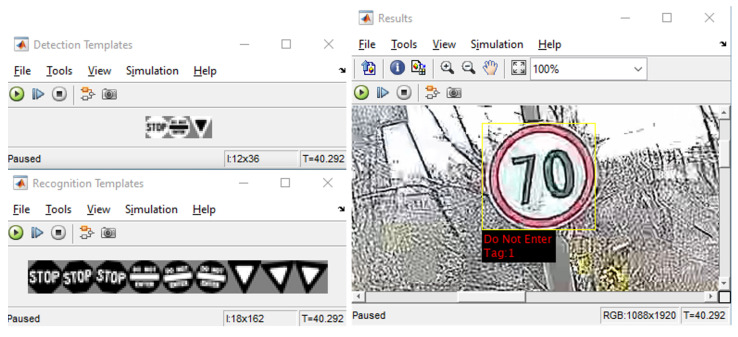
Example of traffic sign detection and classification.

The problem of traffic sign recognition in order to create an automated system was solved using the proposed neural network. The proposed system opens up new possibilities for further research in our future work. The automation of the traffic sign recognition system is becoming increasingly necessary for its use in road traffic. The example of traffic signs classification using CNN is shown in Figure 20. When the “Speed limit” symbol is shown to the camera system, the trained model identifies it and classifies the traffic sign name as “Speed limit”. Classifications and predictions are made in very less time (almost real-time), which benefits drivers. Although the classification of traffic signs has many advantages, there are also some difficulties. For instance, if the traffic sign is covered by trees or any billboard on the side of the road, then it can cause inaccurate traffic sign detection and classification. It may also happen that the vehicle is cruising so fast that the system does not have enough time to correctly recognize the traffic sign. These situations can be very dangerous and lead to traffic accidents.

Various environmental constraints, including lighting, traffic sign distance (the sign is too far away), or shadow, can significantly affect the accurate detection and classification of traffic signs. Therefore, further research in this area is needed.

One of the directions that require further research is traffic light detection, which the industry continues to develop at an ever-increasing pace. Notable examples in this area include recent developments made by several major automotive industry players, whose vehicles already include systems based on either DSRC or image processing for traffic light color recognition and driver alerting.

In our future work, we plan to improve the precision of the proposed neural network for the recognition of traffic signs. We also plan to create a line detection system for automated vehicle driving. The processing of collected data for the infrastructure assessment was not completed in real-time. In our future work, we plan to introduce elements of automatization to the assessment process with the ultimate goal of developing a fully automated system for infrastructure readiness assessment. The research presented in this manuscript is an initial step towards this goal.

Another option for further research is to design a fully automatic road sign recognition system that will work in real-time. This system will use the camera system on the vehicle to detect and recognize traffic signs in real-time. In the event that this system is integrated together with the GPS system, it is also possible to provide the driver with additional practical information about the current restrictions within the current traffic situation on the given road. Based on the comparison of data from GPS and the sign recognition system, this system could warn the driver in the case of disregarding traffic signs. It is worth noting here that the detection and correct classification of live objects is an extremely important aspect of CAV operation. While being beyond the scope of this paper, we aim to incorporate this aspect and address its challenges in our future work as well. 

## Figures and Tables

**Table 1:** The assessment framework—connectivity area.

Indicator	Value	Score
Communication latency	x < 1 ms	1
	1 ms ≤ x < 50 ms	0.75
	50 ms ≤ x < 100 ms	0.5
	x ≥ 100 ms	0
	x < 0.001%	1
Message loss	0.001% ≤ x < 10%	0.5
	x ≥ 10%	0
	x ≥ 1 Gbit/s	1
	24 Mbit/s ≤ x < 1 Gbit/s	0.75
Bitrate per vehicle	8.5 Mbit/s ≤ x < 24 Mbit/s	0.5
	300 kbit/s ≤ x < 8.5 Mbit/s	0.25
	x < 300 kbit/s	0

**Table 2:** The assessment framework—localization area.

Indicator	Value	Score
	x > 20	1
	15 ≤ x < 20	0.75
Average number of satellites	10 ≤ x < 15	0.5
	5 ≤ x < 10	0.25
	x ≤ 5	0
	x ≥ 4	1
	x = 3	0.75
Number of using satellites	x = 2	0.5
	x = 1	0.25
	x = 0	0
	x < 0.1 m	1
GNSS lateral localization error	0.1 m ≤ x ≤ 0.2 m	0.5
	x > 0.2 m	0

**Table 3:** The assessment framework—object detection distance area.

Indicator	Value	Score
Intersection sight distance	*V*_d_ = 62.5 km/h	
	x ≥ 183 m	1
	148 m ≤ x < 183 m	0.75
	113 m ≤ x < 148 m	0.25
	x < 113 m	0
	*V*_d_ = 112.5 km/h	
	x ≥ 329 m	1
	266 m ≤ x < 329 m	0.75
	203 m ≤ x < 266 m	0.25
	x < 203 m	0
Infrastructure for remote	Yes	1
sensor sharing available	No	0

**Table 4:** The assessment framework—quality of maps area.

Indicator	Value	Score
	Static and dynamic infrastructure is available on the map and available to the CAV. Based on the information on the map the vehicle can perceive microscopic traffic situations in real time.	1
Quality of maps	Digital map with detailed lane information and static road signs is available. Traffic lights, short-term road works and variable message signs have to be recognized by AVs.	0.75
	Digital map is available but vehicle has to recognize lane geometry and/or road signs.	0.25
	No digital map is available. The vehicle has to recognize road geometry and traffic signs on its own.	0

**Table 5:** The assessment framework—machine-readable signage area.

Indicator	Value	Score
	x ≥ 99%	1
Precision of horizontal signage detection	90% ≤ x < 99%	0.5
	80% ≤ x < 90%	0.25
	x < 80%	0
Precision of vertical	x ≥ 98%	1
signage detection	x < 98%	0

**Table 6:** DOP value rating [34].

DOP Value	Rating
<1	Ideal
1–2	Excellent
2–5	Good
5–10	Moderate
10–20	Fair
>20	Poor

**Table 7:** GPS FIX status enumeration and technique accuracy [34,38,39].

FIX Quality	Technique	Accuracy (m)
1	GPSFix	15
2	DGPS	0.1
3	PPSFix	<0.03
4	RTK Fixed	0.01–0.02
5	RTK Float	0.75–0.2

**Table 8:** Performance indicator standards for *SINR* [42].

Range (dB)	Category
10 to 30	Excellent
3 to 10	Good
0 to −3	Fair
−20 to −3	Poor

**Table 9:** Performance indicator standards for *RSRP* [46].

Range (dBm)	Category
−80 to −44	Excellent
−90 to −80	Good
−100 to −90	Fair
−110 to −100	Poor
−140 to −110	Very Poor

**Table 10:** Performance indicator standards for *RSRQ* [42].

Range (dB)	Category
−10 to −3	Excellent
−12 to −10	Good
−14 to −12	Fair
−17 to −14	Poor
−20 to −17	Very Poor

**Table 11:** CQI-MCS mapping for LTE rel.12 and beyond [52].

CQI	Modulation	Code Rate	Bits per RE
1	QPSK	0.0762	0.1524
2	QPSK	0.1885	0.377
3	QPSK	0.4385	0.877
4	QPSK	0.3691	1.4764
5	QPSK	0.4785	1.914
6	QPSK	0.6016	2.4064
7	16QAM	0.4551	2.7306
8	16QAM	0.5537	3.3222
9	16QAM	0.6504	3.9024
10	64QAM	0.7539	4.5234
11	64QAM	0.8525	5.115
12	64QAM	0.6943	5.5544
13	64QAM	0.7783	6.2264
14	64QAM	0.8634	6.9072
15	64QAM	0.9258	7.4064

**Table 12:** Specifications of OmniVision OV10640 camera system.

Camera System Parameter	Specification
Resolution	1280 (H) × 1080 (V)
Mega Pixels	1.3 MP
Supply Voltage	1.7 to 3.47 V
Frame Rate	60 fps
Pixel Size	4.2 μm × 4.2 μm
Dynamic Range	120 dB
Sensitivity	8.4 V/lux-s
SNR	41.5 dB

**Table 13:** The classes in the image dataset.

Number of Class	Class Specification	The Overall Number of Data	Training Dataset	Testing Dataset	Validation Dataset
1	Ahead only	1670	1003	500	167
2	Turn center ahead	1670	1003	500	167
3	Turn right ahead	1670	1003	500	167
4	The one-way traffic	1670	1003	500	167
5	The stop sign	1670	1003	500	167
6	Give away	1670	1003	500	167
7	The priority road	1670	1003	500	167
8	The pedestrians	1670	1003	500	167
9	The cyclists	1670	1003	500	167
10	The motorbikes	1670	1003	500	167
11	The scooters	1670	1003	500	167
12	Road closed	1670	1003	500	167
13	Passing prohibited	1670	1003	500	167
14	No entry	1670	1003	500	167
15	Speed limit	1670	1003	500	167
16	No right turn sign	1670	1003	500	167
17	No center turn sign	1670	1003	500	167
18	Two-way traffic ahead	1670	1003	500	167
19	The passenger cars	1670	1003	500	167
20	The Vans/trucks	1670	1003	500	167

**Table 14:** CNN layers.

Layers	Description of Layers
Conv2D_1	32 filters with dimensions 5 × 5, the output is a feature map with dimensions 32 × 32 × 32
Conv2D_2	32 filters with dimensions 5 × 5, the output is a feature map with dimensions 32 × 32 × 32
MaxPooling_1	Filter size 2 × 2, the output is a feature map with dimensions 32 × 16 × 16
Dropout_1	50% neuron shutdown, the output is a 32 × 16 × 16 feature map
Conv2D_3	64 filters with dimensions 3 × 3, the output is a feature map with dimensions 64 × 16 × 16
Conv2D_4	64 filters with dimensions 3 × 3, the output is a feature map with dimensions 64 × 16 × 16
MaxPooling_2	Filter size 2 × 2, the output is a feature map with dimensions 32 × 8 × 8
Dropout_2	50% neuron shutdown, the output is a 32 × 8 × 8 feature map
Flatten_1	4096 neurons
Dense_1	256 neurons
Dropout_3	50% neuron shutdown
Dense_2	20 neurons

**Table 15:** Results of image classification.

Number of Class	Precision (%)	Recall (%)	F1 Score (%)
1	99.8	100	99.9
2	100	100	100
3	100	100	100
4	100	100	100
5	100	100	100
6	100	100	100
7	99.6	100	99.8
8	98.4	97.8	98.1
9	85.5	85.2	85.3
10	86.4	87.4	86.9
11	96.4	96.2	96.3
12	99.6	99.8	99.7
13	100	99.6	99.8
14	99.6	100	99.8
15	100	99.8	99.9
16	100	100	100
17	100	100	100
18	100	99.6	99.8
19	100	100	100
20	100	100	100

## Data Availability

The data presented in this study are available on request from the corresponding author.

## Abbreviations

The following abbreviations are used in this manuscript:

ADS	Automated Driving Systems
CAM	Cooperative Awareness Messages
CCAM	Cooperative, Connected and Automated Mobility
CAVs	Connected and Automated Vehicles
CNN	Convolutional Neural Network
CRS	Cell Reference Signal
CQI	Channel Quality Indicator
CV2X	Cellular Vehicle-to-everything
DGPS	Differential Global Positioning System
DOP	Dilution of Precision
EARFCN	E-UTRA Absolute Radio Frequency Channel Number
GNSS	Global Navigation Satellite Systems
GPS	Global Positioning System
HDOP	Horizontal Dilution of Precision
LTE	Long-Term Evolution
ODDs	Operational Design Domains
PDOP	Position Dilution of Precision
RF	Radio Frequency
RI	Rank Indicator
RSRP	Reference Signal Received Power
RSRQ	Reference Signal Received Quality
RSSI	Received Signal Strength Indicator
RTK	Real Time Kinematic
SBC	Single-Board Computer
SD	Sight Distance
SINR	Signal Interference Noise Ratio
SPS	Standard Positioning Service
TDOP	Time Dilution of Precision
UE	User Equipment
VDOP	Vertical Dilution of Precision
VLC	Visible Light Communication
V2I	Vehicle-to-Infrastructure
V2X	Vehicle-to-everything
